# Exploring osteochondral damage patterns in acute patellar dislocation: insights into morphological associations and risk factors

**DOI:** 10.1038/s41598-024-57363-w

**Published:** 2024-03-20

**Authors:** Yu Gao, Chunxiao Wei, Modi Yang

**Affiliations:** 1 Department of Orthopedics, China-Japan Union Hospital, Jilin University, 126 Xiantai Street, Changchun, 130033 People’s Republic of China; 2 Department of Neurology and Neuroscience Center, The First Hospital of Jilin University, Jilin University, Changchun, People’s Republic of China

**Keywords:** Acute patellar dislocation, osteochondral damage, MRI, Anatomy, Trauma

## Abstract

Osteochondral damage (OD) is a significant outcome following acute patellar dislocation (APD), yet the factors contributing to its susceptibility remain unclear. The primary objective of this study was to assess the association between demographic characteristics, patellofemoral (PF) joint morphology, and the occurrence of OD. A retrospective analysis identified 74 patients with APD who underwent treatment in our unit between 2019 and 2022. All patients received MRI within a week of injury to assess OD, subsequently categorized according to the injury pattern. The Caton-Deschamps index (CDI), tibial tuberosity-trochlear groove distance (TT-TG), lateral trochlear inclination (LTI), sulcus angle (SA), patellar width (PW), patellar thickness (PT), and femoral condyle geometry were calculated from the MRI scans and compared between groups. The findings revealed that OD predominantly manifested in the lateral femoral condyle (LFC) region and the medial patella (MP) region. In our patient cohort, this study identified a significant association between sulcus angle and the incidence of OD in both MP and LFC regions. Additionally, a significant correlation was discerned between skeletal maturity and the incidence of OD in the LFC region within demographic characteristics.

## Introduction

The incidence of acute patellar dislocation (APD) in young, active individuals is nearly 30 per 100,000 person-years, frequently causing damage to structures within the patellofemoral (PF) joint^1–3^. Osteochondral damage (OD) is a severe consequence of APD, linked to knee joint pain and reduced quality of life^4,5^. Following APD, shear forces can induce OD in the lateral femoral condyle and medial patellar region^6,7^. Due to its limited healing capacity, OD tend to worsen over time, increasing the risk of secondary osteoarthritis^8,9^. Therefore, the presence of OD holds crucial implications for both the acute management and long-term clinical outcomes of APD patients.

There is considerable variability in the reporting rate of OD in APD-treated patients, with rates determined by arthroscopy and magnetic resonance imaging (MRI) ranging from 15 to 73%^10–12^. Similarly, the risk factors for OD development in APD patients are not well established. Some studies on APD injury patterns have indicated an association between medial patellofemoral ligament (MPFL) tears and OD^13,14^. Complete MPFL tears are more likely to be associated with osteochondral lesions in the lateral femoral condyle region than partial MPFL tears^15^. Another study also suggested an association between chondral defects of the patella and the geometry (shallow trochlea and patella alta) of the PF joint^16^.

Given the uncertainty in the existing literature, it is crucial to elucidate the prevalence of OD in APD patients and its connection to pathoanatomy. Moreover, understanding the factors contributing to OD susceptibility is vital for developing a predictive model for APD, with significant implications for acute management, surgical decisions, and long-term clinical outcomes in APD patients. Therefore, this study aimed to characterize the location and incidence of OD in APD patients, with a secondary goal of evaluating the association between PF joint morphology and OD.

## Materials and methods

### Patients

The study protocol was approved by the Ethics Committee of the China-Japan Union Hospital of Jilin University and was conducted according to the principles of the Declaration of Helsinki. Written informed consent was obtained from all participants. The study exclusively encompassed individuals diagnosed with APD, commonly referred to as first-time patellar dislocation.

Inclusion criteria were as follows: (1) the typical history and clinical examinations comprised knee hemarthrosis, edema, tenderness upon palpation of the femoral condyle and medial parapatellar structures, and the presence of the apprehension sign; (2) MRI revealed evidence of bone bruising affecting the medial patella and/or the lateral femoral condyle; (3) MRI demonstrated MPFL tears, characterized by edema at the site of injury, with or without evidence of disrupted fibers; (4) MRI was performed within one week of the injury.

Exclusion criteria comprised recurrence or congenital patellar dislocations, prior surgery on the affected knee, lower limb impairment, or past knee injuries. Patients with PF joint osteoarthritis were also excluded.

### MRI technique

The Magnetom Symphony Syngo MR A30 (Siemens, Erlangen, Germany) was used for the MRI, and all of the patients were positioned with their knees fully extended. The following sequences were obtained: a transverse fat-saturated proton-density weighted fast spin-echo imaging sequence (repetition time/echo time, 4500 ms/33 ms; flipangle, 150°; field of view, 160 mm × 160 mm; section thickness, 3.0 mm) was followed by a coronal fat-saturated proton density (2000/15; field of view, 150 mm; matrix, 320 × 224 pixels; slice thickness, 3.0 mm; skip, 0.3 mm), a sagittal proton density (1950–2766/14; field of view, 140–150 mm; matrix range, 320–384 × 192–224 pixels; slice thickness, 3.0 mm; skip, 0.3 mm), a sagittal fat-saturated proton density (2650–4366/13–16; field of view, 140–150 mm; matrix range, 256–384 × 224–256; slice thickness, 3.0 mm; skip, 0.3 mm), and a sagittal T2 (3700–4766/80–87; field-of-view range, 140–150 mm; matrix range, 320–384 × 224; slice thickness, 3.0 mm; skip, 0.3 mm).

### MRI evaluation

Two experienced orthopedic surgeons, each possessing 10 and 15 years of clinical expertise in the realm of sports medicine, independently analyzed the MRI images. They were unaware of any prior imaging interpretations. The readers were blinded to patient information and reviewed all images randomly. Following our established criteria for OD, each orthopedic surgeon independently assessed the images to ascertain the presence of OD. Their findings were initially documented, and in cases of divergent opinions, the images underwent further review to achieve consensus. The average of the two measurements was computed for PF joint morphology following independent assessments by two readers. The interobserver reliability was assessed by calculating the intraclass correlation coefficient (ICC) for each examined parameter.

### Measurements of OD and PF joint morphology

**Figure 1:**
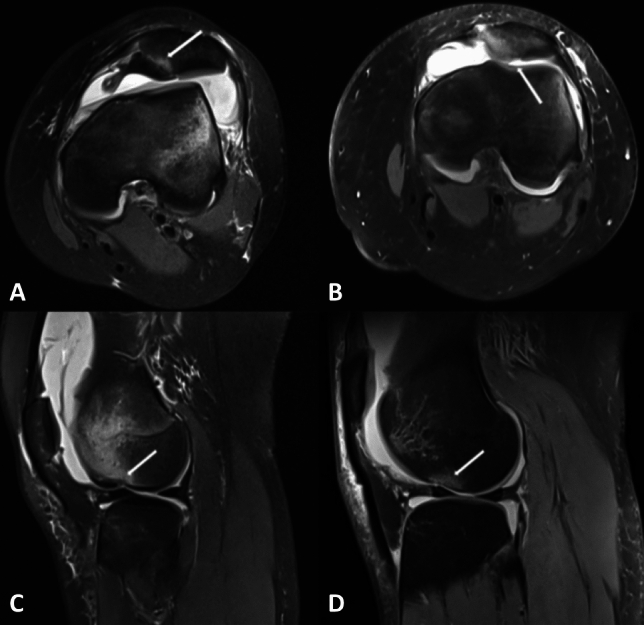
(**A,B**) Depict the axial MRI scans illustrating osteochondral damage to the medial patella (white arrow). (**C,D**) Illustrating osteochondral damage to the lateral femoral condyle (white arrow).

OD was discerned through MRI, and categorized as either damage osteochondral (labeled as “D”) or a normal osteochondral (labeled as “N”). The regions affected by OD were delineated into the lateral femoral condyle (LFC) and medial patella (MP). Manifestations of osteochondral damage encompassed partial-thickness chondral defects (< 100% of the articular cartilage thickness), full-thickness chondral defects (involving intact subchondral bone), osteochondral lesions (involving an underlying cortical defect), and avulsion fractures^17,18^. Examples are depicted in Fig. 1.

**Figure 2:**
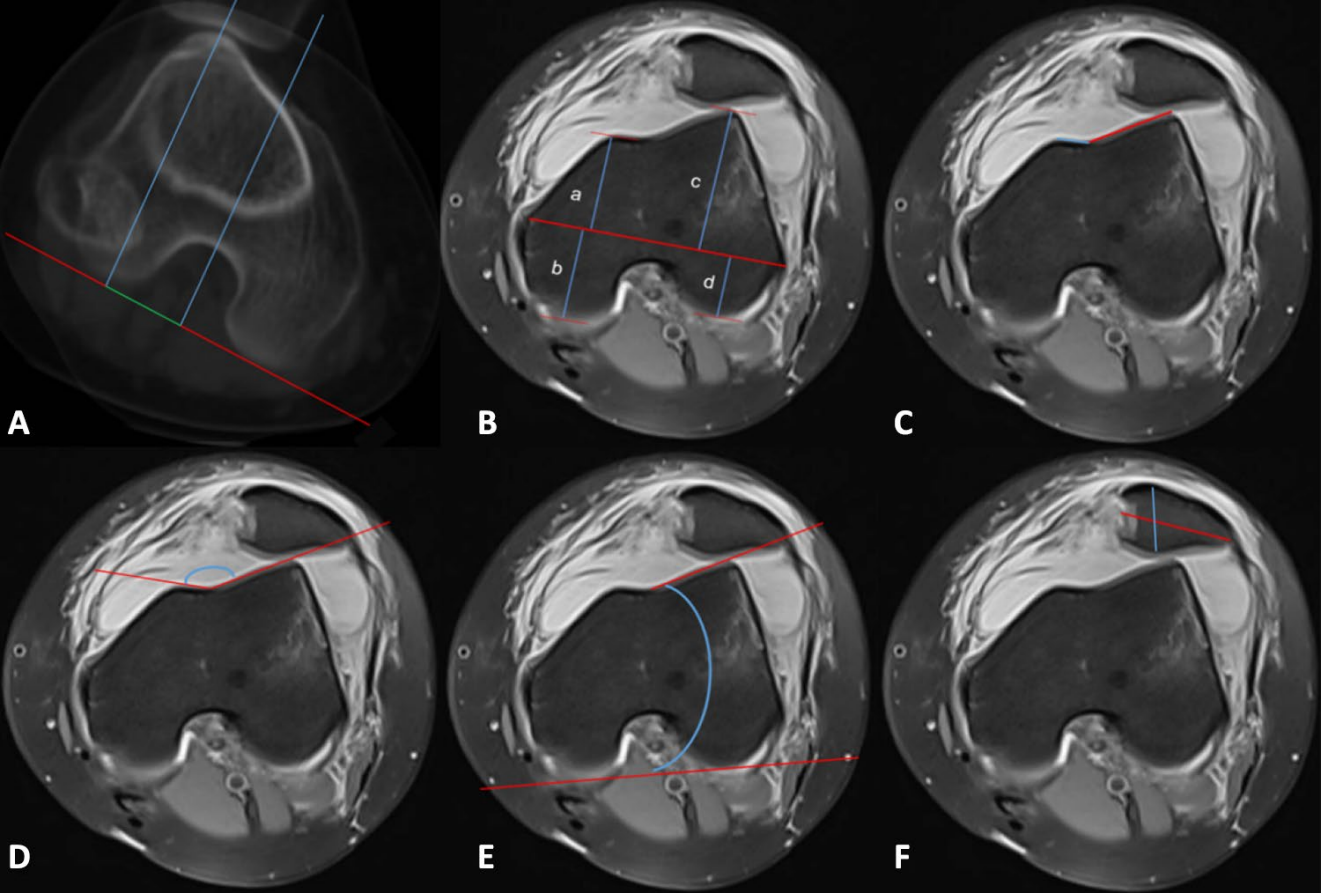
Morphological measurements of the trochlear and patellar regions were conducted as follows: (**A**) TT-TG measurement; (**B**) condylar length measurement in the following regions: (a) anteromedial condyle, (b) posteromedial condyle, (c) anterolateral condyle, (d) posterolateral condyle; (**C**) Measurement of medial and lateral trochlear facet lengths; (**D**) sulcus angle measurement; (**E**) LTI measurement; (F) patellar width and thickness measurement.

Sagittal and axial images were employed for the assessment of PF morphology. To analyze the geometry of the articulating joint, angles and distances were gauged up to the furthest cartilaginous point. Measurements of parameters including the Caton-Deschamps index (CDI)^19^, tibial tuberosity-trochlear groove distance (TT-TG)^20^, patellar width and thickness^16^, medial and lateral trochlear facet length^21^, patellar inclination angle (LTI)^22^, and sulcus angle^23^ were executed following established protocols in the literature. The anatomical epicondylar axis (AEA), a reliable reference on the distal femur, facilitated the measurement of femoral condyle and trochlea morphology in the transverse plane^24^. The femoral condyle was partitioned into four quadrants for a more comprehensive evaluation of the correlation between PF geometry and OD. The vertical distance from the highest point of the condylar cortex to the AEA was measured to determine the lengths of the anterior and posterior condyles. Examples are depicted in Fig. 2.

### Statistical analysis

Statistical analysis was conducted using SPSS 22.0 (IBM SPSS Statistics, Version 22.0). Descriptive statistics were provided for continuous variables (mean and standard deviation) and categorical variables (counts and proportions). Group differences were assessed using independent samples t-tests and chi-square tests. Influential factors were initially explored through one-way analysis, and those demonstrating significance (*p* < 0.05) were incorporated into binary logistic regression analysis. The predictive accuracy of the logistic regression models was evaluated via diagnostic receiver operating characteristic (ROC) curves, with associated areas under the ROC curves (AUC) values. The chosen level of significance was *p* < 0.05.

## Results

The study encompassed 74 participants diagnosed with APD, boasting an average age of 19.74 ± 9.41 years. The cohort comprised 38 males and 36 females, with 44 left knee joints and 30 right knee joints; furthermore, 38.3% exhibited open epiphysis. The prevailing site for OD manifested in the LFC (70%), succeeded by the MP (62%). The ICC values for the measurements surpassed 0.90, indicative of robust concordance.

### Osteochondral damage in lateral femoral condyle region

Within the LFC region, Group D exhibited notably elevated sulcus angle (159.53° ± 16.2° vs. 150.30° ± 12.7°, *P* = 0.012) and significantly reduced anteromedial condyle (21.87 mm ± 3.3 mmvs. 24.47 mm ± 4.3 mm, *P* = 0.013) compared to Group N. Additionally, a majority of closed epiphysis were observed in group D (40.9% vs. 69.2%, *p* = 0.023); however, no significant distinctions surfaced in other demographic parameters such as age and sex (Table 1).

**Table 1:** Results of demographic and geometric measurements in LFC region.

	Osteochondral damage		
Variable	Normal (n = 22)	Damage (n = 52)	*P* value
Age, years	17.68 ± 7.2	20.62 ± 10.2	0.223
Female, n (%)	11 (50)	27 (51.9)	0.880
CDI	1.22 ± 0.2	1.15 ± 0.2	0.107
TT-TG, mm	17.25 ± 2.6	17.88 ± 3.2	0.412
LTI angle, °	12.04 ± 4.6	12.05 ± 6.0	0.993
Sulcus angle, °	150.30 ± 12.7	159.53 ± 16.2	0.012*
Patella thickness, mm	20.44 ± 5.5	20.68 ± 3.6	0.832
Patella width, mm	40.51 ± 5.4	40.78 ± 4.3	0.822
ALC length, mm	37.0 ± 3.1	37.41 ± 3.3	0.660
PLC length, mm	31.56 ± 3.1	31.59 ± 2.9	0.971
AMC length, mm	24.47 ± 4.3	21.87 ± 3.3	0.013*
PMC length, mm	26.54 ± 3.9	27.72 ± 3.0	0.162
LTF length, mm	23.93 ± 3.5	24.4 ± 3.2	0.531
MTF length, mm	12.00 ± 3.1	11.92 ± 3.3	0.922
Closed epiphyses, n (%)	9 (40.9)	36 (69.2)	0.023*

*P* values were calculated via t-test and Chi-square test.

*CDI* Caton-Deschamps index, *TT-TG* tibial tuberosity-trochlear groove distance, *LTI* lateral patellar inclination, *ALC* anterolateral condyle, *PLC* posterolateral condyle, *AMC* anteromedial condyle, *PMC* posteromedial condyle, *MTF* medial trochlear facet, *LTF* lateral trochlear facet.

*Statistically significant at *p* < 0.05.

**Figure 3:**
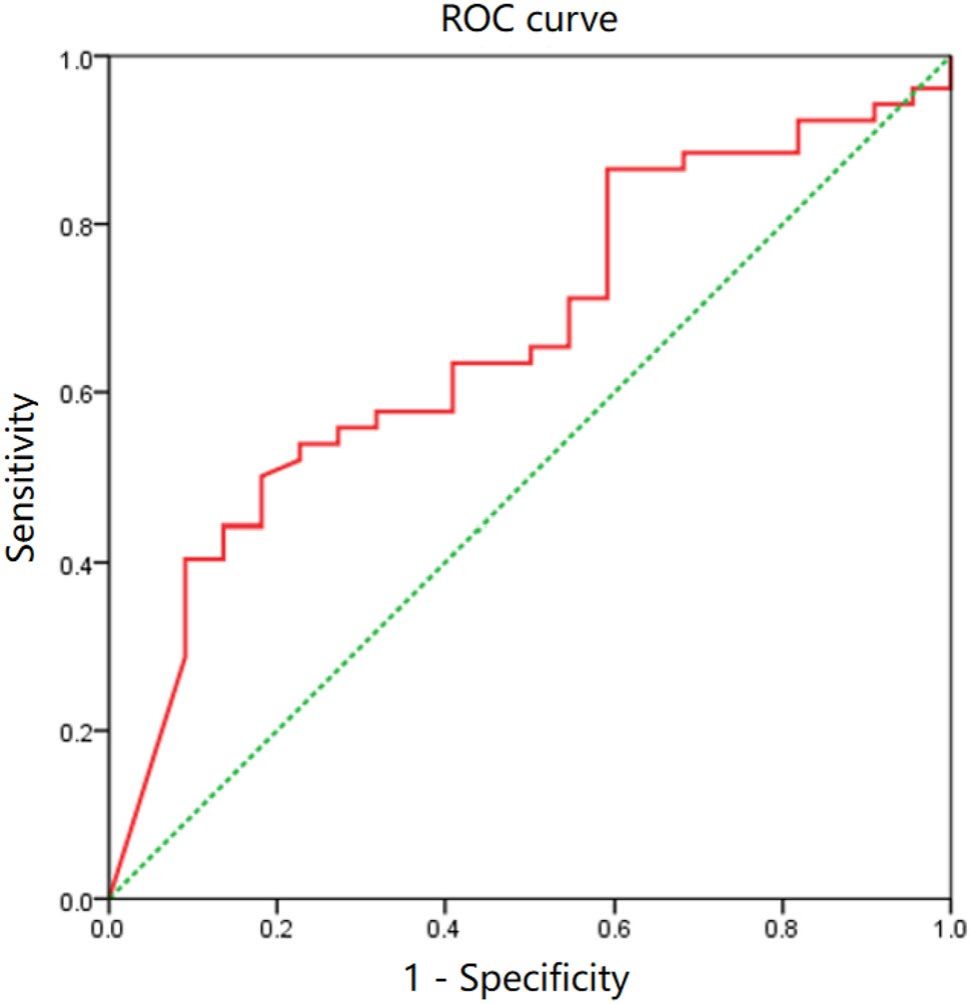
A moderate area under the curve of 0.667 is indicated by the area under the ROC curve plot for the sulcus angle.

To investigate factors influencing OD occurrence, binary logistic regression was employed to establish a correlation model between OD (normal or damaged) and all analyzed parameters (Table 2). Results indicated a significant association between closed epiphyses and OD development (*p* = 0.040). Patients with closed epiphyses were 3.068 times more likely to experience OD than those with open epiphyses. Additionally, the sulcus angle exhibited a significant correlation with OD development in patients (*p* = 0.037). With each 1° increase in the sulcus angle, the likelihood of OD development increased by 1.04 times. The ROC curve for the sulcus angle model was constructed (Fig. 3), yielding a modest AUC of 0.667 (*p* = 0.024, 95% CI 0.536–0.797).

**Table 2:** Association between variables and OD in LFC region.

Variable	B	*P* value	OR	95% CI
Closed epiphyses, n (%)	1.121	0.040*	3.068	1.051–8.960
Sulcus angle, °	0.039	0.037*	1.040	1.002–1.079

*P* values and CIs were calculated via binary logistic regression.

*Statistically significant at *p* < 0.05.

### Osteochondral damage in medial patella region

In the MP region, the D group exhibited an extended medial trochlear facet (12.52 mm ± 3.7 mm vs. 10.99 mm ± 2.8 mm, *p* = 0.049) and an elevated sulcus angle (160.54° ± 15.3° vs. 150.61° ± 14.6°, *P* = 0.008) compared to the N group. Nevertheless, no discernible disparities in demographic characteristics were identified between groups N and D (Table 3).

**Table 3:** Results of demographic and geometric measurements in MP region.

	Osteochondral damage		
Variable	Normal (n = 28)	Damage (n = 46)	*P* value
Age, years	20.21 ± 10.7	19.46 ± 8.6	0.793
Female, n (%)	14 (50)	24 (52.2)	0.856
CDI	1.21 ± 0.2	1.15 ± 0.1	0.132
TT-TG, mm	18.04 ± 3.3	17.02 ± 2.8	0.980
LTI angle, °	12.68 ± 5.1	11.65 ± 5.9	0.449
Sulcus angle, °	150.61 ± 14.6	160.54 ± 15.3	0.008*
Patella thickness, mm	19.54 ± 3.3	21.26 ± 4.7	0.090
Patella width, mm	41.06 ± 3.6	40.49 ± 5.2	0.612
ALC length, mm	37.45 ± 3.7	37.20 ± 3.0	0.755
PLC length, mm	31.34 ± 3.6	31.73 ± 2.6	0.591
AMC length, mm	24.30 ± 3.7	22.70 ± 4.8	0.109
PMC length, mm	26.93 ± 3.1	27.64 ± 3.5	0.373
LTF length, mm	24.26 ± 3.8	24.33 ± 3.0	0.928
MTF length, mm	10.99 ± 2.8	12.52 ± 3.7	0.049*
Closed epiphyses, n (%)	16 (57.1)	29 (63.0)	0.614

*P* values were calculated via t-test and Chi-square test.

*CDI* Caton-Deschamps index, *TT-TG* tibial tuberosity-trochlear groove distance, *LTI* lateral patellar inclination, *ALC* anterolateral condyle, *PLC* posterolateral condyle, *AMC* anteromedial condyle, *PMC* posteromedial condyle, *MTF* medial trochlear facet, *LTF* lateral trochlear facet.

*Statistically significant at *p* < 0.05.

**Figure 4:**
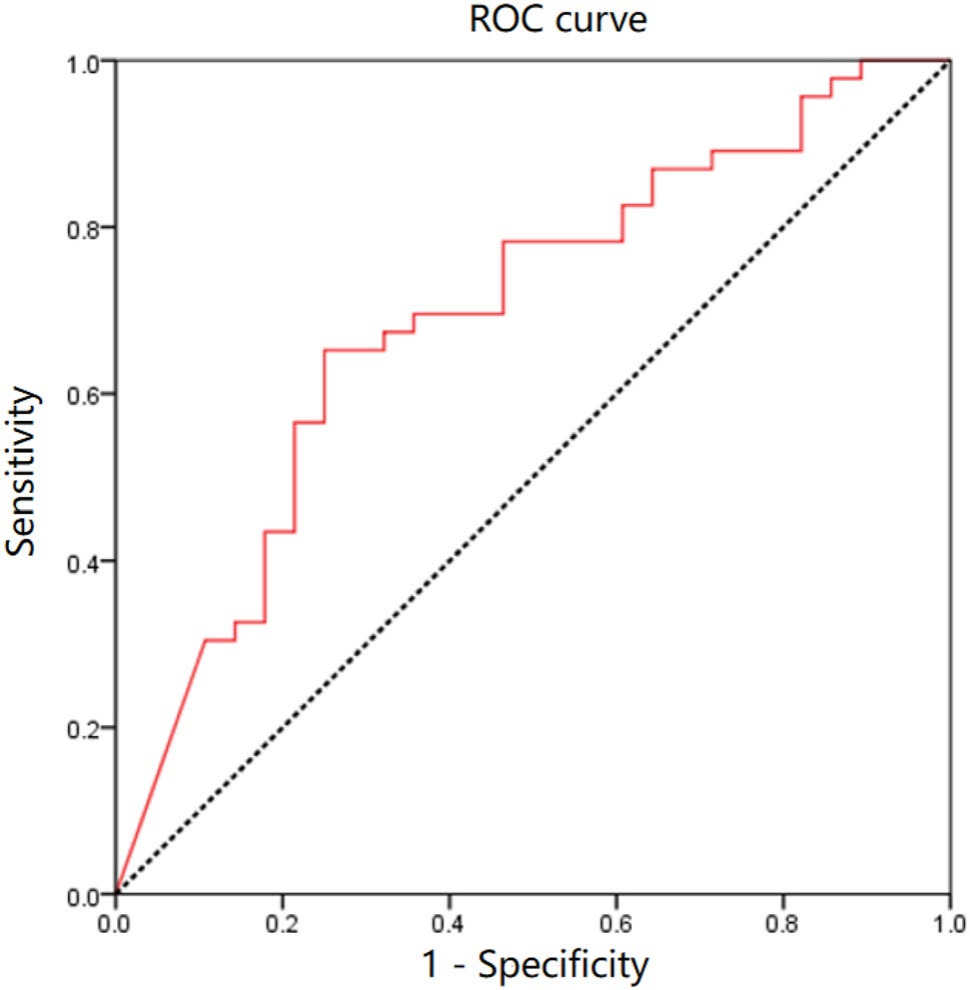
A moderate area under the curve of 0.695 is indicated by the area under the ROC curve plot for the sulcus angle.

In a more in-depth investigation of susceptibility factors for OD occurrence, binary logistic regression demonstrated a noteworthy association between the sulcus angle and OD incidence (Table 4). With each 1° increment in sulcus angle, the likelihood of OD occurrence increased by 1.053 times. No other demographic characteristics or PF morphology variables exhibited statistically significant associations with OD occurrence. The ROC curve for the sulcus angle model was established (Fig. 4), yielding a modest AUC of 0.695 (*p* = 0.005, 95% CI 0.570–0.819).

**Table 4:** Association Between variables and OD in MP region.

Variable	B	*P* value	OR	95% CI
MTF length, mm	0.216	0.051	1.204	1.020–1.509
Sulcus angle, °	0.052	0.006*	1.053	1.015–1.093

*P* values and CIs were calculated via binary logistic regression.

*Statistically significant at *p* < 0.05.

## Discussion

The primary discovery of this investigation reveals morphological distinctions in the PF joint among patients afflicted by OD when compared to control subjects without OD. Additionally, variations in patient demographic characteristics were observed among different groups. Subsequent multifactorial regression analyses unveiled the intricate connections between these variables and the onset of OD. An elevated sulcus angle exhibited a significant correlation with osteochondral damage in both the LFC and MP regions. Nevertheless, skeletal maturity demonstrated a significant correlation solely with OD in the LFC region. This suggests meticulous consideration of geometric irregularities in the PF joint and epiphyseal development when caring for patients with APD. Additionally, there should be risk stratification for patients necessitating early surgical intervention to prevent the progression of patellofemoral arthritis.

The reported rates of OD in our study cohort, namely 70% in the lateral femoral condyle region and 62% in the medial patella region, align with the broad spectrum reported in previous studies (15%–73%)^2,12,25^. Despite the elevated probability of concurrent osteochondral damage in APD patients. Oftentimes, the management of OD proves highly ineffective due to diagnostic oversights, consequently culminating in secondary osteoarthritis of the PF joint^26–28^. As cartilage is not visible on X-ray, relying solely on X-ray assessments is inadequate for patients with APD. Given the elevated prevalence of OD, employing MRI emerges as a more conclusive and comprehensive diagnostic tool^29,30^. This modality can not only detect ligament and osteochondral injuries but also assess the patient's anatomical irregularities, thereby aiding in informed decision-making.

The primary mechanism underlying OD in the LFC and MP regions is predominantly ascribed to mutual impingement during patellar dislocation and reset^31,32^. Our investigation revealed a higher incidence of OD in the LFC region compared to the MP region (70% vs. 62%). Our hypothesis posits that the heightened occurrence of OD in the LFC region may arise not only from impingement in the medial patellar area but also in connection to torsional forces on the knee joint, akin to the mechanism observed in anterior cruciate ligament rupture. Vollnberg et al.^33^ identified a connection between acute patellar dislocation (APD) and patellar cartilage injury in around 71% of their subjects, while Guerrero et al.^34^ reported that APD-related patellar osteochondral injuries manifested in 49% of patients, with a higher predilection in males. However, our findings did not reveal a correlation between age, gender, and the incidence of OD. Subsequent binary regression analysis unveiled that skeletal maturity independently correlated with the incidence of OD in the LFC region. Individuals with closed epiphyses exhibit an approximately threefold higher likelihood of experiencing osteochondral damage in the LFC region compared to those without epiphyseal closure. Plausible explanations include the superior elasticity of the epiphyseal plate and ligamentous stretch in patients with unclosed epiphyses, contributing to shock absorption and cushioning against a portion of the patellar impact force. Conversely, patients with skeletal maturity experience heightened shear and impact forces on the LFC region, rendering them more susceptible to osteochondral injury.

Subsequently, we delved into the association between OD and PF joint geometry. Within the LFC region, the damaged group exhibited a larger sulcus angle and a shorter AMC length compared to the normal group. In the MP region, statistical analysis revealed an increase in both sulcus angle and MTF length within the damage group. Nevertheless, upon amalgamating all variables in a regression analysis, only the sulcus angle demonstrated a significant association with the incidence of OD. The current literature presents a controversy regarding the relationship between PF pathoanatomy and OD. Seeley and co-works^2^ reported that 72% of patients with OD displayed aberrant PF geometry, encompassing features like patella alta and trochlear dysplasia. Conversely, certain studies have indicated an absence of correlation between OD and conditions like patella alta and trochlear dysplasia^25,35^. Within our study cohort, binary logistic regression analysis led us to the conclusion that an elevated sulcus angle independently correlated with an increased risk of developing OD in patients with APD, supported by a moderate AUC in the ROC analysis. Trochlear dysplasia was quantified by measuring the sulcus angle through MRI. In contrast to other analogous studies, where trochlear dysplasia was assessed using the Dejour method with low intra- and inter-observer reliability^20,36^. Furthermore, while the lengths of AMC and MTF did not independently influence the occurrence of OD, they did indicate the presence or severity of trochlear dysplasia^37,38^. Consequently, it is imperative to exercise vigilance in patients with APD, particularly in those exhibiting an elevated sulcus angle.

Osteochondral damage demonstrates a robust correlation with an unfavorable patient prognosis and the progression of advanced osteoarthritis^39^. In case of combined full-thickness chondral defect, osteochondral lesion, and fracture may require timely surgical intervention. Significantly, even the co-occurrence of partial-thickness chondral defect should be regarded seriously, given that patients are typically children and adolescents; early intervention holds pivotal importance in curbing potential irreversible complications in the future. It is important to acknowledge that this study has certain limitations. The study primarily utilized MRI for assessing OD following APD, neglecting intraoperative assessment, which could result in potential inaccuracies and overlook other significant measurements, such as valgus-valgus alignment in long-leg X-rays. Secondly, the exclusion of certain patients lacking imaging data might have led to the omission of eligible individuals, introducing selection bias and limiting the overall sample size of this study. Additionally, owing to the limited number of cases, we lacked the statistical power to conduct valid calculations for grading the severity of OD. Consequently, this study primarily concentrated on determining the presence or absence of OD and identifying the specific sites where OD occurred. The spectrum of OD in this study was defined to encompass partial-thickness chondral defect, full-thickness chondral defect, osteochondral lesion, and fractures. Our future research endeavors, aimed at achieving greater statistical efficacy, hold potential in elucidating predictors of OD by integrating larger sample sizes and utilizing WORMS scores to classify the severity of OD.

## Conclusions

Patients with APD are susceptible to combined OD, which is more likely to occur in the lateral femoral condyle region than in the medial patellar region. This study demonstrated that an increased sulcus angle was significantly associated with the incidence of OD in a cohort of patients with APD. In addition, skeletal maturity was also significantly associated with the occurrence of OD at the lateral femoral condyle site.

## Data Availability

The datasets generated during and/or analysed during the current study are not publicly available due to privacy of patient information, but are definitely available from the corresponding author if requested. All requests are greeted.
